# Quack leptin

**DOI:** 10.1186/1471-2164-15-551

**Published:** 2014-07-03

**Authors:** Miriam Friedman-Einat, Eyal Seroussi

**Affiliations:** Institute of Animal Science, The Volcani Center, Rishon Le-Zion, Israel

**Keywords:** Obese gene, Lipogenesis, Domestic fowls

## Abstract

**Background:**

A *LEP* transcript up-regulated in lungs of ducks (*Anas platyrhynchos*) infected by avian influenza A virus was recently described in the *Nature Genetics* manuscript that reported the duck genome. In vertebrates, *LEP* gene symbol is reserved for leptin, the key regulator of energy balance in mammals*.*

**Results:**

Launching an extensive search for this gene in the genome data that was submitted to the public databases along with duck genome manuscript and extending this search to all avian genomes in the whole-genome shotgun-sequencing database, we were able to report the first identification of coding sequences capable of encoding the full leptin protein precursor in wild birds. Gene structure, synteny and sequence-similarity (up to 54% identity and 68% similarity) to reptilian leptin evident in falcons (*Falco peregrinus* and *cherrug*), tits (*Pseudopodoces humilis*), finches (*Taeniopygia guttata*) and doves (*Columba livia*) confirmed that the bird leptin was a true ortholog of its mammalian form. Nevertheless, in duck, like other domestic fowls the *LEP* gene was not identifiable.

**Conclusion:**

Lack of the *LEP* gene in poultry suggests that birds that have lost it are particularly suited to domestication. Identification of an intact avian gene for leptin in wild birds might explain in part the evolutionary conservation of its receptor in leptin-less fowls.

## Correspondence

### Background

The duck (*Anas platyrhynchos*) genome and transcriptome were recently reported in *Nature Genetics* [1] as part of an investigation of immune-related genes implicated in the response to infection by avian influenza virus A. Using deep sequencing, the authors compared the lung transcriptomes of control and *H5N1-*infected ducks and used the gene symbol *LEP* to describe a transcript that was upregulated in the infected ducks. In vertebrates, this gene symbol is reserved for leptin, the key regulator of energy balance in mammals; however, the avian ortholog has never been established.

### Leptin in poultry research

An entry in the Gene database [Gene ID: 373955] is set aside for the chicken (*Gallus gallus*) leptin gene. The lack of a nucleotide sequence for this entry reflects its complex history, having been cloned and its sequence then retracted [2–5]. After removing the *Bos taurus* sequences that contaminated the first submission of the chicken genome project and the EST database [6], it was finally established that no close ortholog of mammalian leptin is present in this genome. However, the obvious importance of identifying a master gene that controls appetite and fattening in poultry promoted cloning of mammalian-like leptins in turkey (*Meleagris gallopavo*, [GenBank: AAC32381], 95% identity to mouse leptin) and duck ([GenBank: AAT38807], 99% identity to mouse leptin). In the turkey genome housed in the ENSMBL database, there are neither annotations for *LEP* nor murine-like leptin sequences in its build; hence, like chickens, turkeys lack leptin.

### Results and discussion

#### Synteny confirms leptin in birds

**Figure 1:**
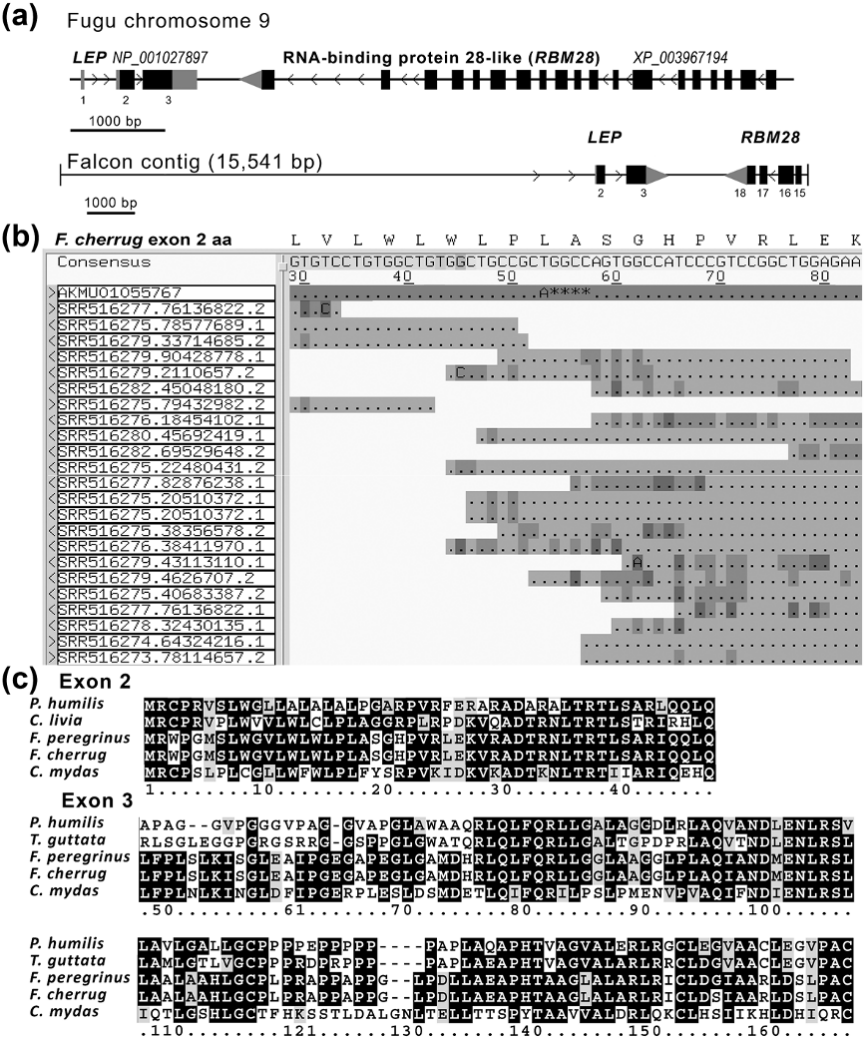
**Synteny, shotgun assembly, and sequence alignments of avian leptin-like genes. (a)** Comparison of genomic region of fugu (*Takifugu rubripes*) *LEP* based on the NCBI Sequence Viewer display, and falcon contig assembly [GenBank: AKMT01018335–6, AKMU01055766–7]. Gene structures are drawn to scale shown by the bar below. Black and gray boxes represent translated and untranslated regions (UTRs) of exons, respectively. When the exact transcription termination site is not characterized, large gray arrowheads at the 3′ UTRs indicate the direction of transcription, which is generally indicated by small arrowheads on the intron delineations. Gene identification and exon numbers are given above and below the gene depictions, respectively. Exon numbers for falcon *RBM28* follow their numbering in the orthologous rat gene. **(b)** Identification of errors in the genome submission of falcon based on alignment with individual reads from Sequence Read Archive (SRA). Reads were located by BLASTN search of the SRA database, downloaded with their quality information (FASTQ format), and assembled using GAP5 software [8]. The relevant protein sequence is added above the contig editor output for a region of low coverage within the second *LEP* exon. The contig editor shows quality values by gray scale and discrepancies between the sequences and the consensus are highlighted by a base symbol. The cutoff option was not turned on and therefore low quality (dark gray) bases that were manually trimmed are not displayed. Individual reads and the mapping template (AKMU01055767) are identified on the left. A base substitution and 4-base deletion (A****) are denoted on the mapping template, which is the first read below the consensus line. **(c)** Amino acid sequences of leptin-like genes identified in the WGS database of birds:tit (*Pseudopodoces humilis*, [GenBank: HG425120]), dove (*Columba livia*, [GenBank: HG425123]), falcons (*Falco peregrinus*, [GenBank: HG425121]; and *F. cherrug*, [GenBank: HG425122]), and finch (*Taeniopygia guttata*, [GenBank: XP_004175839]) were compared with turtle *LEP* (*Chelonia mydas*, [GenBank: KB475412]). Box coloration follows the legend of Figure 2.

The typical structure of the leptin gene include 3 exons: a non-coding exon followed by large intron 1, a second exon harboring the translation-initiation codon close to the splicing acceptor site, and a third large exon that encodes most of the protein (e.g. human Gene ID: 3952, fugu, *Takifugu rubripes*, Figure 1a). Based on a short contig (1482 bp) of a whole-genome shotgun sequencing (WGS) project, a partial gene capable of encoding the third exon of a leptin-like protein has recently been annotated in *Taeniopygia guttata* [Gene ID: 101233729], suggesting that leptin is expressed in the zebra finch. A BLASTP search using the putative 115-aa polypeptide encoded by this exon against the NR database indicated that the reptilian green sea turtle (*Chelonia mydas*) leptin was its closest ortholog, with 34% identity and 52% similarity (Figure 1c), while mouse leptin was more distant with 31% identity and 47% similarity. We hypothesized that if leptin is indeed present in birds, it would have been revealed in other avian WGS projects. Indeed, TBLASTN against the WGS database with taxid restricted to *Aves* revealed that the gene may be present in falcons (*Falco peregrinus* and *F. cherrug*; [GenBank: AKMT01018335 and AKMU01055767], respectively), tits (*Pseudopodoces humilis*, [GenBank: ANZD01014665, ANZD01014667]) and doves (*Columba livia*, AKCR01028475). The falcon sequences were 99.6% syntenic [7] and we therefore assembled the contigs of both species together (15,541 bp, also including [GenBank: AKMT01018336 and AKMU01055766], Figure 1a) using GAP4 and 5 software [8], and incorporating additional reads in critical regions from the Sequence Read Archive Nucleotide BLAST (Figure 1b). This revealed coding exons fitting the typical leptin gene structure and capable of encoding a full-length leptin-like 166-aa precursor with 52% identity and 69% similarity of *F. cherrug* to the turtle leptin (Figure 1c). Moreover, the 3′-neighboring gene of the falcon leptin showed 56% identity and 68% similarity to rat RNA-binding motif protein 28 (*RBM28,* [Gene ID: 312182]). Local *LEP*–*RBM28* synteny is conserved and observed in fish (e.g. fugu, [Gene ID: 101064097]) and mammals (Figure 1a), and thus strongly indicating that these sequences are orthologous to the mammalian leptin.The tit contigs were GC-rich (68%) with highly repetitive GC elements and we were unable to combine them; nevertheless, both coding exons corresponding to the typical leptin structure were observable. These exons were capable of encoding a full-length leptin-like 161-aa precursor with 36% identity and 57% similarity to the turtle leptin (Figure 1c). Further search of the WGS database revealed similarity to single exons: the previously annotated exon 3 for zebra finch and a novel match to exon 2 for dove. We extended the detected dove contig with reads [SRA: SRR511892.31385855, SRR511913.3134902] and found the initiation codon of a typical structure of leptin exon 2 capable of encoding 48 aa of the 5′ end of a leptin-like precursor with 56% identity and 72% similarity to turtle leptin (Figure 1c, [GenBank: HG425123]). Evidence for expression of this exon was provided by a single read derived from racing-liver RNA-Seq library prepared from poly-A + RNA [SRA: SRR521362.22831237.2]. This read represented a spliced transcript of 2 non-coding exons that preceded the first coding exon, in agreement with the sequence of the introns and canonical splice sites evident in the genomic submission (GenBank: AKCR01028476).

#### Leptin remains unidentifiable in domestic fowls

Examination of the recently submitted duck genome annotations revealed no gene with *LEP* as its symbol and no gene annotated as leptin. Moreover, BLASTN search of the WGS database using “duck leptin” [GenBank: AAT38807] or any of the novel leptin-like bird proteins described here indicated no significant similarity to leptin in this genome submission. Thus, we conclude that this gene may be also missing in duck. It is expected of the of the editorial process of a high ranking journal to ensure that when seeking a fast impact, genome publications would not turn into lists of unverified gene symbols that no one actually reads. It is further recommended that authors who deposited erroneous sequences of murine-like leptins for birds in sequence databases [GenBank: AAC32380, AAC60368, AAL35557, AAT38807, O42164, O93416] caution users of the possibility of sequence contamination. It should be also noted that 11 GenBank mRNA submissions of fish leptins with >98% identity to the mouse transcript should be similarly annotated [GenBank: DQ784814-6, AY497007, AY547279, AY547322, AY551335-9].

Moreover, a large volume of misinformation may have been generated as these murine-like leptins were the basis for studies without prior knowledge of leptin’s activity in the targeted species, including reports of the expression of the erroneous leptin gene product at the mRNA and protein levels (e.g. [9–16]). These leptins were reported to attenuate appetite, or affect other parameters related to the control of energy balance when administered to chickens [17–20], chick embryos [21–23], ovarian [24] and hepatoma [25] cells in culture or skeletal bones in an *ex vivo* model system [26].

#### Recent findings

**Figure 2:**
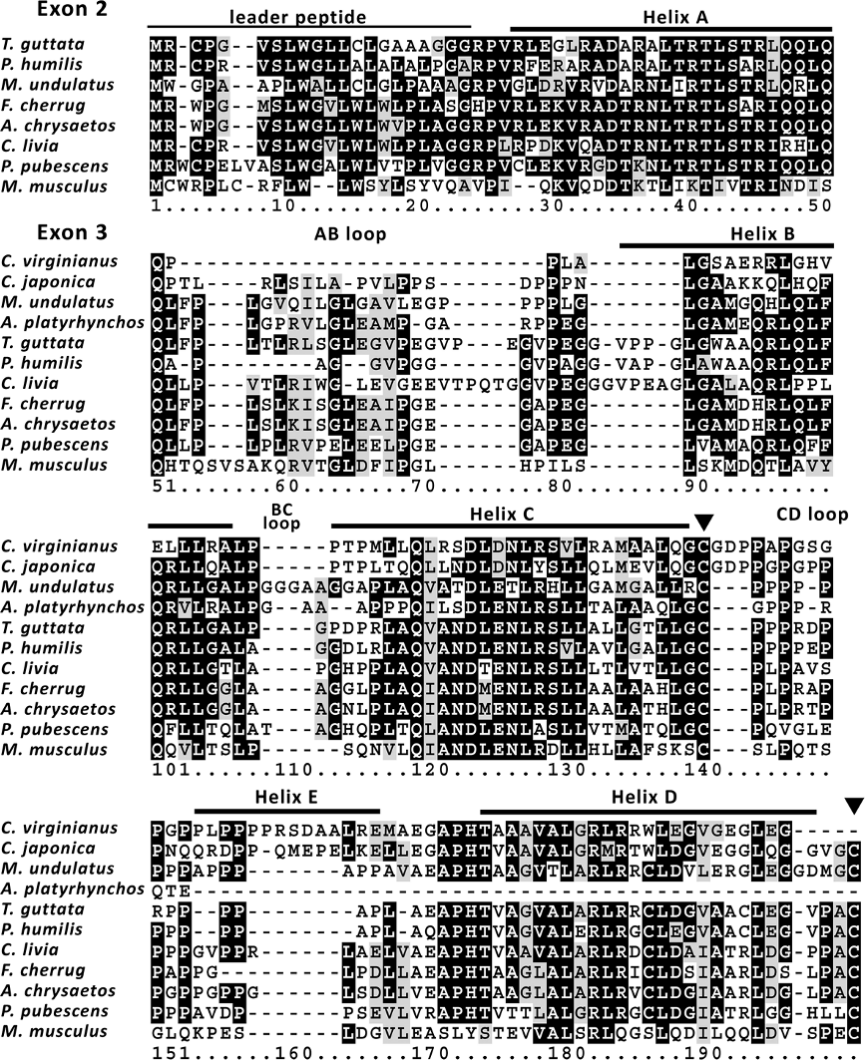
**The amino-acid sequence of mouse leptin (** ***Mus musculus*** **, [GenBank: NP_032519]) was aligned with leptin and** ***LEP*** **-like sequences of birds identified in the WGS and SRA databases: zebra finch (** ***Taeniopygia guttata*** **, [GenBank: AFK25168]); Tibetan ground tit (** ***Pseudopodoces humilis*** **, [GenBank: HG425120]); budgerigar (Melopsittacus undulates, [GenBank: AHZ86931]); falcon (** ***Falco cherrug*** **, [GenBank: HG425122]); golden eagle (** ***Aquila chrysaetos Canadensis*** **, derived from [GenBank: JDSB01143511, SRR1016445.84242652, SRR1016445.37770192, JDSB01163119, SRR1017148.40189562]; dove (** ***Columba livia*** **[GenBank: HG797022]); downy woodpecker (** ***Picoides pubescens*** **, derived from [GenBank: JJRU01076739]); northern bobwhite quail (** ***Colinus virginianus*** **, derived from [GenBank: AWGU01372785]); Japanese quail (** ***Coturnix japonica*** **, derived from [GenBank: ERR125582.247893.2, DRR002300.424669919.1, DRR002301.19253882.1, DRR002301.124106485.1, DRR002301.44847625.1]); and the mallard duck (** ***Anas platyrhynchos*** **, derived from [GenBank: SRR040307.6134664, SRR797835.67134665.2, SRR040316.4927613, SRR797835.67134665.1]).** Dashes indicate gaps introduced by the alignment program. Identical and similar amino-acid residues in at least three or six sequences are indicated by a black and gray background, respectively. White boxes indicate non-conservative amino-acid changes between the proteins. The signal peptide and structural elements, helixes and loops [28] are denoted above the alignment. The two conserved cysteines forming a lasso knot [30] are indicated by black arrowheads. Duck’s genomic sequence was confirmed using previously described procedures [28]; DNA was extracted from frozen mallard duck purchased from a local husbandry (Levin, Kfar Baruch, Israel) and nucleotide sequence was determined by capillary sequencing of the 81 bp product amplified using PCR primers (F, 5′-CAGCTTTTCCAGCGCGTC-3; R, 5′-GAGGTTCTCCAGGTCGCTTA-3′).

While this work was under consideration 3 reports describing avian leptins were submitted and published [27–29], including indication that is based on a single RNA-seq read for leptin-like transcript in the duck [27]. We used the sequence information from this read and the read from this fragment opposite end, to design a pair of PCR primers which bridged the sequence gap between these reads. The PCR protocol applied was adapted for amplification of leptin GC rich sequences [28]. DNA sequencing of the resulting PCR product confirmed the existence of leptin-like sequence orthologous to the sequence of the last exon of other avian leptins (Figure 2) in the duck genome. However, analysis of the genomic raw deep-sequencing data [BioProject: PRJNA46621] was hampered by existence of similar repetitive sequence structures; and we were able to extend this sequence only towards the 5′. We detected no reads that could extend the 3′ with sequence coding for valid cysteine knot motif that is typical of all leptins [30]. Furthermore, analyzing the raw RNA-seq data [BioProjects: PRJNA194464, PRJNA188394] revealed transcription matching the repetitive sequence structures but no additional reads for the duck leptin-like sequence described here could be identified (data not shown). Detection of leptin syntenic genes like *miR129–1* favors the possibility that the leptin gene may also exist in ducks [29]. Hence, the existence of fully functional leptin gene in the duck remains an open question.

Further BLASTN and TBLASTN searches of the WGS database using the novel avian leptin sequences revealed indications for existence of leptin in additional bird species. These include woodpecker, eagle and quails (Figure 2). Protein motifs typical of leptin were identified and annotated including leader peptide, 4-helix bundle structure and cysteine knot (Figure 2). While the leptin gene of woodpecker was apparent on an unplaced genomic scaffold [GenBank: JJRU01076739] the gene of golden eagle was much obscure. The eagle’s first coding exon (exon 2) was intact in a WGS contig [GenBank: JDSB01143511]. However, de-novo assembly of genomic raw deep-sequencing data [BioProject: PRJNA222866] was unable to extend the sequence of the last-exon-like structure [GenBank: JDSB01163119]. Yet, all the putative motifs encoded by the highly (89% identity and 91% similarity) orthologous falcon leptin gene were assembled to form disordered palindromic and repetitive contigs containing also the leptin’s syntenic gene *RBM28*. Such structures were also typical for the duck (data not shown). Bobwhite quail was the first *galliforme* with a partial exon 3 like sequence observable in a contig assembly of the WGS effort of this quail (Figure 2, [GenBank: AWGU01372785]). We used this sequence as a template for a BLAST search of the deep-sequencing data deposited for the Japanese quail in the SRA database and the related WGS assembly. The leptin gene was not identifiable in the latter, however we were able to download and assemble the matching SRA sequence reads (Figure 2), which correspond to an intact exon 3 structure. We repeated the sequence searches against the chicken genome and confirmed that even this *galliforme LEP*-like sequence is not detectable in *Gallus gallus*, in agreement with the observation that administration of a leptin antagonist had no effect on appetite and body growth of layer chickens [31]. We could not associate any ESTs or RNA-seq reads to the quails’ leptin-like genes and moreover the role of the leptin signaling pathway may differ in *galliformes* [28]. This hypothesis may also be related to the finding that the hunger hormone ghrelin [32], which is predominantly synthesized in the gastrointestinal tract in chickens and mammals, has been reported to have an opposite effect on appetite in chickens compared to mammals [33, 34]. Hence, galliformes provide a unique model system to decipher an alternative control mechanism of energy homeostasis and we intend to further study this in the Japanese quail.

## Conclusions

The absence of a leptin gene in genomes related to domestic fowls seems incompatible with the presence of the leptin receptor gene, which has been cloned in chicken [35], turkey [36] and duck [1]. Herein we report the first identification of coding sequences capable of encoding the full leptin protein precursor in birds. Identification of an intact avian gene for leptin might explain in part the evolutionary conservation of its receptor in *Aves*. The loss of leptin in the lineage of domestic fowls suggests that relaxing the control of appetite made these birds particularly suited to domestication.

### Methods

#### Comparative sequence analysis

For the characterization of leptin genes not yet annotated in the avian genomes assemblies, sequence homology searches were carried out in different, publicly available database (NCBI: NR, WGS, SRA; and Ensembl) using the BLAST family of programs. Relevant sequence entries were downloaded with their quality information (FASTQ format), and reassembled using the GAP5 software [8]. The amino acid sequences were aligned using CLUSTALW (http://www.genome.jp/tools/clustalw/) with the default parameters and the GONNET matrix; and colored using the BOXSHADE program (http://www.ch.embnet.org/software/BOX_form.html).

#### Sequence data accessions

The annotated sequences are available in GenBank under accessions HG425120-3.

## References

[1] Huang Y, Li Y, Burt DW, Chen H, Zhang Y, Qian W, Kim H, Gan S, Zhao Y, Li J, Yi K, Feng H, Zhu P, Li B, Liu Q, Fairley S, Magor KE, Du Z, Hu X, Goodman L, Tafer H, Vignal A, Lee T, Kim KW, Sheng Z, An Y, Searle S, Herrero J, Groenen MA, Crooijmans RP (2013). The duck genome and transcriptome provide insight into an avian influenza virus reservoir species. Nat Genet.

[2] Friedman-Einat M, Boswell T, Horev G, Girishvarma G, Dunn IC, Talbot RT, Sharp PJ (1999). The chicken leptin gene: Has it been cloned?. Gen Comp Endocr.

[3] Sharp PJ, Dunn IC, Waddington D, Boswell T (2008). Chicken leptin. Gen Comp Endocr.

[4] Pitel F, Faraut T, Bruneau G, Monget P (2010). Is there a leptin gene in the chicken genome? Lessons from phylogenetics, bioinformatics and genomics. Gen Comp Endocr.

[5] Prokop JW, Duff RJ, Ball HC, Copeland DL, Londraville RL (2012). Leptin and leptin receptor: analysis of a structure to function relationship in interaction and evolution from humans to fish. Peptides.

[6] Carre W, Wang XF, Porter TE, Nys Y, Tang JS, Bernberg E, Morgan R, Burnside J, Aggrey SE, Simon J, Cogburn LA (2006). Chicken genomics resource: sequencing and annotation of 35,407 ESTs from single and multiple tissue cDNA libraries and CAP3 assembly of a chicken gene index. Physiol Genomics.

[7] Zhan XJ, Pan SK, Wang JY, Dixon A, He J, Muller MG, Ni PX, Hu L, Liu Y, Hou HL, Chen YP, Xia JQ, Luo Q, Xu PW, Chen Y, Liao SG, Cao CC, Gao SK, Wang ZB, Yue Z, Li GQ, Yin Y, Fox NC, Wang J, Bruford MW (2013). Peregrine and saker falcon genome sequences provide insights into evolution of a predatory lifestyle. Nat Genet.

[8] Bonfield JK, Whitwham A (2010). Gap5–editing the billion fragment sequence assembly. Bioinformatics.

[9] Ashwell CM, Czerwinski SM, Brocht DM, McMurtry JP (1999). Hormonal regulation of leptin expression in broiler chickens. Am J Physiol-Reg I.

[10] Richards MP, Ashwell CM, McMurtry JP (1999). Analysis of leptin gene expression in chickens using reverse transcription polymerase chain reaction and capillary electrophoresis with laser-induced fluorescence detection. J Chromatogr A.

[11] Ashwell CM, Richards MP, McMurtry JP (2001). The ontogeny of leptin mRNA expression in growing broilers and its relationship to metabolic body weight. Domest Anim Endocrin.

[12] Taouis M, Dridi S, Cassy S, Benomar Y, Raver N, Rideau N, Picard M, Williams J, Gertler A (2001). Chicken leptin: properties and actions. Domest Anim Endocrin.

[13] McMurtry JP, Ashwell CM, Brocht DM, Caperna TJ (2004). Plasma clearance and tissue distribution of radiolabeled leptin in the chicken. Comp Biochem Phys A.

[14] Dridi S, Buyse J, Decuypere E, Taouis M (2005). Potential role of leptin in increase of fatty acid synthase gene expression in chicken liver. Domest Anim Endocrin.

[15] Rao KQ, Xie JJ, Yang XJ, Chen L, Grossmann R, Zhao RQ (2009). Maternal low-protein diet programmes offspring growth in association with alterations in yolk leptin deposition and gene expression in yolk-sac membrane, hypothalamus and muscle of developing Langshan chicken embryos. Brit J Nutr.

[16] Ngernsoungnern P, Sartsoongnoen N, Prakobsaeng N, Chaiyachet OA, Chokchaloemwong D, Suksaweang S, Ngernsoungnern A, Chaiseha Y (2012). Plasma leptin concentrations during the reproductive cycle in the native Thai chicken (*Gallus domesticus*). Anim Reprod Sci.

[17] Dridi S, Raver N, Gussakovsky EE, Derouet M, Picard M, Gertler A, Taouis M (2000). Biological activities of recombinant chicken leptin C4S analog compared with unmodified leptins. Am J Physiol-Endoc M.

[18] Denbow DM, Meade S, Robertson A, McMurtry JP, Richards M, Ashwell C (2000). Leptin-induced decrease in food intake in chickens. Physiol Behav.

[19] Li RJ, Hu Y, Ni YD, Xia D, Grossmann R, Zhao RQ (2011). Leptin stimulates hepatic activation of thyroid hormones and promotes early posthatch growth in the chicken. Comp Biochem Phys A.

[20] Paczoska-Eliasiewicz HE, Proszkowiec-Weglarz M, Proudman J, Jacek T, Mika M, Sechman A, Rzasa J, Gertler A (2006). Exogenous leptin advances puberty in domestic hen. Domest Anim Endocrin.

[21] Su L, Rao K, Guo F, Li X, Ahmed AA, Ni Y, Grossmann R, Zhao R (2012). In ovo leptin administration inhibits chorioallantoic membrane angiogenesis in female chicken embryos through the STAT3-mediated vascular endothelial growth factor (VEGF) pathway. Domest Anim Endocrin.

[22] Liu P, Hu Y, Grossmann R, Zhao R (2013). In ovo leptin administration accelerates post-hatch muscle growth and changes myofibre characteristics, gene expression and enzymes activity in broiler chickens. J Anim Physiol an N.

[23] Hu Y, Zhang R, Zhang YH, Li J, Grossmann R, Zhao RQ (2012). In ovo leptin administration affects hepatic lipid metabolism and microRNA expression in newly hatched broiler chickens. J Anim Sci Biotechno.

[24] Sirotkin AV, Grossmann R (2007). Leptin directly controls proliferation, apoptosis and secretory activity of cultured chicken ovarian cells. Comp Biochem Phys A.

[25] Cassy S, Derouet M, Crochet S, Dridi S, Taouis M (2003). Leptin and insulin downregulate leptin receptor gene expression in chicken-derived leghorn male hepatoma cells. Poultry Sci.

[26] Mauro LJ, Wenzel SJ, Sindberg GM (2010). Regulation of chick bone growth by leptin and catecholamines. Poultry Sci.

[27] Prokop JW, Schmidt C, Gasper D, Duff RJ, Milsted A, Ohkubo T, Ball HC, Shawkey MD, Mays HL, Cogburn LA, Londraville RL (2014). Discovery of the elusive leptin in birds: identification of several ‘missing links’ in the evolution of leptin and its receptor. Plos One.

[28] Friedman-Einat M, Cogburn LA, Yosefi S, Hen G, Shinder D, Shirak A, Seroussi E (2014). Discovery and characterization of the first genuine avian leptin gene in the rock dove (*Columba livia*). Endocrinology.

[29] Huang G, Li J, Wang H, Lan X, Wang Y (2014). Discovery of a novel functional Leptin Protein (LEP) in Zebra Finches: evidence for the existence of an authentic avian leptin gene predominantly expressed in the brain and pituitary. Endocrinology.

[30] Haglund E, Sulkowska JI, He Z, Feng GS, Jennings PA, Onuchic JN (2012). The unique cysteine knot regulates the pleotropic hormone leptin. Plos One.

[31] Gertler A, Shinder D, Yosefi S, Shpilman M, Rosenblum CI, Ruzal M, Seroussi E, Freidman-Einat M (2014). Pegylated leptin antagonist with strong orexigenic activity in mice is not effective in chickens. J Exp Biol.

[32] Kojima M, Hosoda H, Date Y, Nakazato M, Matsuo H, Kangawa K (1999). Ghrelin is a growth-hormone-releasing acylated peptide from stomach. Nature.

[33] Kaiya H, Van Der Geyten S, Kojima M, Hosoda H, Kitajima Y, Matsumoto M, Geelissen S, Darras VM, Kangawa K (2002). Chicken ghrelin: Purification, cDNA cloning, and biological activity. Endocrinology.

[34] Saito ES, Kaiya H, Takagi T, Yamasaki I, Denbow DM, Kangawa K, Furuse M (2002). Chicken ghrelin and growth hormone-releasing peptide-2 inhibit food intake of neonatal chicks. European Journal of Pharmacology.

[35] Horev G, Einat P, Aharoni T, Eshdat Y, Friedman-Einat M (2000). Molecular cloning and properties of the chicken leptin-receptor (CLEPR) gene. Mol Cell Endocrinol.

[36] Richards MP, Poch SM (2003). Molecular cloning and expression of the turkey leptin receptor gene. Comp Biochem Phys B.

